# Prevalence of health-risk behaviours among Canadian post-secondary students: descriptive results from the National College Health Assessment

**DOI:** 10.1186/1471-2458-13-548

**Published:** 2013-06-06

**Authors:** Matthew YW Kwan, Guy EJ Faulkner, Kelly P Arbour-Nicitopoulos, John Cairney

**Affiliations:** 1 Department of Family Medicine, McMaster University, 175 Longwood Road South Suite 201a, Hamilton, ON, L8P 0A1, Canada; 2 Faculty of Kinesiology and Physical Education, University of Toronto, Toronto, Canada

**Keywords:** Multiple health behaviours, University students, University health

## Abstract

**Background:**

It is important to understand health-risk behaviours among young adults, as modifications in this can enhance and lessen the risk of chronic illness later in life. The purpose of the current study was to determine the prevalence of a broad range of health-risk behaviours among post-secondary students from across Canada, and to determine whether institutional variability exists in the prevalence of these behaviours.

**Methods:**

Data were collected from 8,182 undergraduate students enrolled in one of eight Canadian post-secondary institutions during the fall or spring of 2009, using the National College Health Assessment (NCHA). The NCHA consists of 60 questions, assessing student health status and engagement in various health behaviours.

**Results:**

Findings show relatively low prevalence in smoking (13.1%) marijuana (17.5%) or other illicit drug use (3.5%), and risky sexual behaviour (12%). Binge drinking, however, was much higher, with nearly 60% of students consuming more than 5 alcoholic drinks in a single occasion during the past 15 days. Similarly, prevalence rates for physical inactivity (72.2%), inadequate sleep (75.6%) and low fruit and vegetable intake (88.0%) were all high among the student population. Results also found that students in smaller institutions exhibited higher rates of inactivity, binge drinking, and marijuana and illicit drug use compared to institutions having a larger student body.

**Conclusion:**

Overall, findings point to the need for more concentrated health promotion campaigns, specifically targeting sleep, fruit and vegetables intake, and greater participation in physical activity. Given evidence of some institutional variability, future efforts are warranted in exploring how best to increase institutional commitment for collecting surveillance data on Canadian post-secondary students.

## Background

Despite health promotion efforts, young adults continue to engage in high rates of health-risk behaviours [1,2]. Many risk factors such as smoking, excessive alcohol consumption, inadequate nutritional status, and low levels of physical activity have direct behavioural links to chronic disease, yet are amenable to change. Modification of these health-risk behaviours can enhance health and lessen the risk of chronic illnesses later in life [2,3].

Evidence suggests that initiation of diseases such as atherosclerosis, obesity, and diabetes related to physical inactivity, is more frequently emerging in the second and third decades of life [4]. Young adults, including post-secondary students, however, do not attribute such health-risk behaviours to the development and progression of these diseases [5]. The transition out of high school is a critical time for which individuals begin to take definitive steps towards independence, and is considered to be the first major transition an individual faces [6]. Together, there is growing recognition that post-secondary students should be a target population for health promotion efforts, including suggestions that their health is an 'important and neglected public health problem’ [1,7,8].

Across Canada and the United States, epidemiological evidence indicates that the prevalence of smoking and binge drinking rapidly increases as the adolescent population moves towards early adulthood [5,9-13]. While the initiation of smoking tends to occur prior to early adulthood, [13] there is research to suggest that the collegiate years are a critical time when many students begin to experiment with smoking. Approximately 25% of post-secondary students are either daily or occasional smokers [14,15]. Likewise, entry into college/university also appears to lend itself to problem drinking, affecting almost all post-secondary campuses [10]. Nearly 70% of post-secondary students reported alcohol use within the last month, [9] with a vast majority of those students reporting at least one session of binge drinking (defined as ≥ 5 drinks in a single occasion) during that time [14,15]. Significant declines in physical activity participation during the transition between high school and post-secondary education have also been evident [16-18].

Given the public health implications of these health-risk behaviours, greater efforts must be taken to prevent their occurrence as Canadian adolescents make their transition into early adulthood. The Canadian Campus Survey [1,11] has been the only focused attempt at estimating the prevalence of health behaviours of the Canadian postsecondary population. However, this survey was restricted to alcohol and drug usage. In 2004, 6,282 full-time university undergraduates from 40 universities completed questionnaires by mail or online. Overall, 18.5% and 6.6% of the students reported consuming 5+ and 8+ drinks on a single occasion, 12.7% were current smokers, and 16.7% had smoked cannabis in the previous 30 days.

Unfortunately, there is no coordinated system for collecting health-related data on post-secondary students in Canada. This is a significant gap because to inform interventions in this setting, a mechanism is required to assess the prevalence and correlates of health behaviours. The information can then in turn, guide intervention prioritization, selection, implementation, and ongoing evaluation and program/health service refinement. There have been some efforts to address this gap. Formed in 1973, the Canadian Association of College and University Student Services (CACUSS) is a professional bilingual association representing and serving those individuals who work in Canadian post-secondary institutions in Student Affairs and Services. The Canadian Organization of University and College Health (COUCH), a Division of CACUSS, is dedicated to improving the health and wellness of college and university communities. In the absence of a Canadian surveillance mechanism, COUCH has advocated for institutions to subscribe to the National College Health Assessment service of the American College Health Association (NCHA-ACHA; for more detail see [13]). Data from one of these institutions has been used to address a range of research questions [19,20].

In 2009, eight Canadian institutions participated in the NCHA-ACHA. The primary purpose of the current study was to examine the prevalence of a broad range of health-risk behaviours among Canadian post-secondary students from these institutions. The secondary purpose was to test for institutional variability in the prevalence of these health-risk behaviours.

## Methods

### Database and sample

Data were collected from 8 English-speaking institutions across 5 provinces in Canada during the fall or spring of 2009, using the NCHA-ACHA. The NCHA consists of 60 questions and approximately 300 items, including assessments of student health status and engagement in a variety of health behaviours; and has been evaluated extensively for reliability and validity in US postsecondary students (for further information, see ACHA [21]). Approximately 10% of the student body from each institution was randomly chosen to receive an invitation by e-mail to participate in the survey. Over a 30-day period, each potential participant received three such invitations to complete an online survey, which was maintained by the ACHA. As incentives, participants were entered into draws to receive gift cards. To obtain a more homogenous group – reflective of the majority of post-secondary students in Canada – only full-time undergraduate students were included in the current analyses. All institutions received approval for administering the survey by their respective University Research Ethics Boards, and the authors received approval from the American College Health Association to conduct the analysis with data from individual institutions.

### Measures

#### Student-demographics

Participants provided demographic information such as age, gender, ethnicity, living situation (e.g., on/off campus/parental home), and year of study.

#### Smoking, marijuana, illicit drugs

Questions related to cigarette smoking, marijuana use, and illicit drug use asked: “*Within the last 30 days*, *on how many days did you use the following*…” Response options ranged from *never used* and *have used but not in the last 30 days* to *used all 30 days*, and were subsequently dichotomized to user (in the past 30 days), or non-user (have not used in the past 30 days).

#### Binge drinking

The question relating to binge drinking asked, “*Within the last 15 days*, *how many times did you have 5 or more drinks in one sitting*?” Respondents indicating that they engaged in one or more sessions of binge drinking during the past 15 days were considered ‘binge drinkers’, and those who did not engage in binge drinking within the past 15 days were considered ‘non-binge drinkers’.

#### Risky sexual behaviour

Participants were asked, “*Within the last 12 months*, *have you experienced the following as a consequences of your drinking*…*had unprotected sex*?” Responses were categorized as yes (they engaged in this health-risk behaviour), or no (for reasons that they do not drink or that they did not engage in this health-risk behaviour).

#### Physical inactivity

Moderate-to-vigorous physical activity (MVPA) behaviours were assessed by two items: “*On how many of the past 7 days did you*: *Do moderate*-*intensity cardio or aerobic exercises* (*caused a noticeable increase in heart rate*, *such as brisk walk*) *for at least 30 minutes*?”; *and* “*Do vigorous*-*intensity cardio or aerobic exercises* (*caused large increase in breathing or heart rate such as jogging*) *for at least 20 minutes*?” Participants answered on a scale from 0 days to 7 days. Consistent with the former Canadian physical activity guidelines that specifies a minimum of four days of either moderate activity or endurance activity, scores of the two items were summed, and reclassified to reflect *insufficiently active* (students that engaged in 3 days or less of MVPA per week) or *sufficiently active* (students that engaged in MVPA for 4 or more days of the week).

#### Lack of fruit and vegetable intake

A single item asked participants to indicate: “*How many servings of fruits and vegetables do you usually have per day*? (*1 serving* = *1 medium piece of fruit*; ½ *cup fresh*, *frozen*, *or canned fruits*/*vegetables*; ¾ *cup fruit*/*vegetable juice*; *1 cup salad greens*; ¼ *cup dried fruits*).” Response options ranged from *0 servings per day*, *1*–*2 servings per day*, *3*–*4 servings per day*, and *5 or more servings per day*. Consistent with previous research, [22] participant responses were dichotomized to reflect *insufficient fruit and vegetable intake* (consuming < 5 servings of fruit and vegetables each day) or *sufficient fruit and vegetable intake* (consuming ≥ 5 or more servings of fruits and vegetables).

#### Inadequate sleep

Participants were asked “*On how many of the past 7 days did you get enough sleep so you felt rested when you woke up in the morning*?” Responses ranged from 0 days to 7 days, and were subsequently dichotomized to reflect either *not having sufficiently enough sleep* (restful on < 4 nights per week) or *having sufficiently enough sleep on most nights of the week* (i.e., ≥ 4 or more nights).

## Results

A total of 10,778 students from across the eight institutions completed the NCHA survey. The final sample size decreased to 8182 following a list wise deletion of participants not meeting the inclusion criteria (see Table 1 for sample characteristics). Participants in the current study were similar to the representative sample included in the 2004 Canadian Campus Survey [1]. Overall, respondents were predominantly female with most living either off-campus with family or on their own, while the year of study was evenly distributed (i.e., 29% first year, 23% fourth year).

**Table 1:** Demographic characteristics of participants

	**Sample total ( *N * = 8182)**	
**Characteristics**	***n***	**%**
Gender		
Female	5542	67.7
Male	2608	31.9
Mean Age (range 18–88)	20.92 ±3.73	
Year of Study		
1st	2354	28.8
2nd	2081	25.4
3rd	1901	23.2
4th or more	1846	22.5
Race or Ethnicity		
White, not Hispanic (includes Middle Eastern)	5169	63.2
Black, not Hispanic	174	2.1
Hispanic or Latino	174	2.1
Asian or Pacific Islander	1854	22.7
Native American	194	2.4
Other	296	3.6
International Student Status	1114	13.7
Living Situation		
Parents/Guardian’s Home	2858	34.9
On Campus	2200	26.9
Off Campus (away from home)	3106	38.0

*Note*: Percentages may not add up to 100 due to incomplete participant data; Values for age: (*n*) = mean and standard deviation.

Overall, the findings show low prevalence in terms of smoking and drug use. Results indicate that only a small proportion of the student population smoked cigarettes (13.1%), used marijuana (17.5%), used other illicit drugs (3.5%), and/or had unprotected sex as a consequence of being intoxicated (12.0%) over the past month. The prevalence of binge drinking, however, was much higher, with nearly 60% of students who reported consuming > 5 alcoholic drinks in a single occasion during the last 15 days. Results also indicated that 72.2% of students were physically inactive (engaged in < 4 days of MVPA) and 75.6% were not getting enough sleep to be rested on ≥ 4 nights each week. Even less encouraging, results show that 88.5% of the student population consumed < 5 servings of fruits and vegetables each day. It is important to note that measures from both physical activity and fruit and vegetable intake likely underestimate the actual prevalence of not meeting current physical activity or fruit and vegetable intake guidelines [23].

In testing for institutional variability, whilst trying to maintain school anonymity, comparisons in the prevalence of health behaviours were made between large (campuses with ≥ 20,000 students) and small institutions (campuses with < 20,000 students). Overall, the results indicate that physical inactivity, binge drinking, marijuana and other illicit drug use, and risky sexual behaviours were all significantly higher among students on smaller campuses (see Table 2). Significant differences also emerged in fruits and vegetables intake, smoking, and insufficient sleep. Further analyses conducted to determine whether there were significant differences between individual institutions within the larger and smaller category of schools. With the exception of illicit drug use – which appears to be consistent across all schools – there appears to be significant differences in health behaviours across different institutions.

**Table 2:** **Prevalence of health**-**risk behaviours among Canadian undergraduates**

	**Total (%)**	**Campus size % (range)**	
	**(*N*** **= 8182)**	**≥20,000 students (*n*** **= 4737)**	**<19,999 students (*n*** **= 3445)**
< 5 daily servings of fruits/vegetables	88.5	88.1	89.1
		(86.8-90.8)^c^	(87.2-92.6)^d^
< 4 times of moderate-to-vigorous PA	72.2	75.8	70.7**
		(71.2-78.1)	(69.1-75.8)^d^
Smoked cigarette(s) in the past 30 days	13.1	12.8	13.5
		(11.8-15.3)^c^	(11.4-18.0)^d^
Binge Drank in the past 2 weeks^a^	59.4	51.1	70.8**
		(43.4-58.3)^c^	(45.1-78.1)^c^
Used marijuana in the past 30 days	17.5	15.3	20.5**
		(14.2-15.8)	(13.2-24.1)^d^
Used illicit drugs in the past 30 days^b^	3.5	2.87	4.47**
		(2.6-3.1)	(2.9-5.1)
Had intercourse while intoxicated in the past 30 days	12.0	9.0	16.7**
		(7.6-10.8)	(8.1-19.4)^d^
Adequate sleep on < 4 nights per week	75.7	75.8^d^	75.6
		(71.2-78.1)	(72.2-81.3)^d^

Note: Prevalence statistics are represented as percentages; There were 4 institutions that were considered large (≥ 20,000 students), and 4 institutions that were considered small <20,000 students).

* Significant differences in campus size (*p* < 0.05).

** Significant differences in campus size (*p* < 0.01).

^a^ Binge drinking characterized as 5 or more drinks in one sitting over the past 2 weeks.

^b^ Illicit drug use characterized as having used an illicit drug other than marijuana over the past 30 days.

^c^ Significant differences between individual institutions (*p* < 0.05).

^d^ Significant differences between individual institutions (*p* < 0.01).

## Discussion and conclusion

Overall, prevalence estimates of health-risk behaviours across the eight Canadian post-secondary institutions ranged from 3.5% in use of illicit drugs, to 88.5% that are consuming less than 5 servings of fruits and/or vegetables each day. Traditionally, there has been a public health focus to reduce “risky health behaviours”, with particular interest directed towards risk factors associated with non-communicable diseases (i.e., smoking, illicit drug use, and binge drinking) [24]. Among these, binge drinking had the highest prevalence (59.4%) in our sample. Although assessed differently, results from the Canadian Campus Survey [1] reported lower prevalence in binge drinking, with 18.5% of their undergraduate sample consuming 5 or more drinks on a single occasion at least twice a month. The prevalence of smoking (12.7% versus 13.1%), cannabis use (16.7% versus 17.5%), and illicit drug use (2.2% versus 3.5%) however, were comparable to findings of the population-based study by Adlaf and colleagues [1]. This may suggest that campuses as a whole are doing a good job of dissuading the use of these substances among post-secondary students. Despite the low prevalence of traditional health-risk behaviours, the majority of students were not engaging in healthy behaviours either. The absence of engaging in these positive health risk behaviours poses its own health a risk, [25] thus was characterized as health-risk behaviours. Findings show that the vast majority of the student population was considered to be physical inactive (72.2%), lacking of sleep (75.7%), and consuming inadequate servings of fruits and vegetables each day (88.5%). Perhaps a shift from the public health perspective is required for administrators at post-secondary institutions. In addition to prevention of prevailing health-risk behaviours, greater investments should be placed towards the promotion of positive health behaviours.

A secondary purpose of the current study was to examine the institutional variability across health-risk behaviours. Significant between-group differences were found based on the basis of campus size. Specifically, institutions that had fewer students (i.e., < 20,000) exhibited higher rates of inactivity, binge drinking, and marijuana and illicit drug use compared to institutions having a larger student body (i.e., ≥ 20,000). Furthermore, there appears to be variability between individual institutions irrespective of the size of the campus. Speculatively, the disparities in health-risk behaviour may be indicative of discrepancies in health-promoting efforts across each campus to reduce the prevalence of such behaviours across the post-secondary institutions. For example, institutions with a smaller student population may have less funding available to build capacity for delivering effective campus-wide health-promotion initiatives in comparison to institutions with a greater student population. Similarly, there might be institutional differences in health promotion efforts, with each school employing different strategies at targeting the health of students. The development of audit tools would be helpful for assessing school level variation in health promotion efforts to confirm such possibilities.

Having a surveillance system such as the NCHA survey that can regularly monitor health-risk behaviours across institutions might make it possible to determine over time the institutions that are successful in changing health behaviours of interest. In turn, this information might pinpoint promising policies or strategies associated with such change, which can then be disseminated nationally. This is the first study to collectively examine the NCHA data from multiple Canadian post-secondary institutions. As such, our study provides a baseline for the prevalence of health-risk behaviours among Canadian post-secondary students. In the absence of a Canadian surveillance mechanism, the US-based NCHA survey seems to be a promising monitoring tool to collect health-related data on Canadian post-secondary students. However, more institutions should be encouraged to participate in future surveys. By reaching a greater number of institutions, collected data will enable opportunities to examine priority health issues affecting the broader Canadian post-secondary population, explore differences between geographic regions, and provide a basis for making comparisons of prevalence and progress to national and provincial norms.

There are some limitations to the study. First, as there were only eight Canadian post-secondary institutions that participated in the 2009 NCHA survey, the reported prevalence may not be representative of the larger, Canadian post-secondary student population. Second, only 10% of the student body from each of the participating institutions was randomly invited to participate in the survey; thus there is the potential for non-response bias. Third, there were seasonal differences in terms of the administration of the NCHA across the institutions. Two of the institutions administered the survey during the spring of 2009, while the remaining six administered the survey during the fall of 2009. It may therefore be possible for these seasonal differences to influence students' responses to some of the measured health-risk behaviours. Finally, while self-report instruments such as the NCHA are useful tools for gathering public health data, [26,27] the nature of such data may be influenced by response bias. Additionally, some measures within the NCHA instrument are less than optimal. In particular, physical activity is not assessed in a way that allows interpretation in terms of ascertaining whether individuals are meeting current physical activity guidelines.

Overall, our study provides preliminary population-level data on the prevalence of common health-risk behaviours among the Canadian post-secondary population. Our findings point to the need for more concentrated health promotion campaigns targeting health promoting behaviours; including obtaining more sleep, consuming more fruit and vegetables, and greater participation in physical activity. Future efforts are warranted in exploring how best to increase post-secondary institutional commitment to collecting surveillance data. Consideration should also be given to the development and validation of a Canadian Postsecondary Health Surveillance System with more rigorous sampling procedures to ensure representativeness.

## Competing interest

The authors declare that they have no competing interests.

## Authors’ contributions

All the authors (MYK, GEF, KPA, & JC) contributed to the conception of the study and made significant contribution to the final paper. MYK, GEF, & KPA, were responsible for data analysis and contributed to the initial draft of the paper. All authors have read and approved the final manuscript. MYK is guarantor of the paper.

## Pre-publication history

The pre-publication history for this paper can be accessed here:

http://www.biomedcentral.com/1471-2458/13/548/prepub

## References

[B1] Adlaf EM, Demers A, Gliksman L Canadian campus survey 2004 2005 Toronto, ON: Centre for Addiction and Mental Health

[B2] Dawson KA Schneider MA Fletcher PC Bryden PJ Examining gender differences in the health behaviours of Canadian university students J R Soc Promote Health 2007 127 1 38 44 10.1177/1466424007073205 17319316

[B3] Poortinga W Perceptions of the environment, physical inactivity and obesity Soc Sci Med 2006 63 11 2835 46 10.1016/j.socscimed.2006.07.018 16952415

[B4] Leslie E Sparling PB Owen N University campus settings and the promotion of physical activity in young adults: lessons from research in Australia and the USA Health Education 2001 101 3 116 25 10.1108/09654280110387880

[B5] Poortinga W The prevalence and clustering of four major lifestyle risk factors in an English adult population Prev Med 2007 44 2 124 8 10.1016/j.ypmed.2006.10.006 17157369

[B6] Brooks JH DuBois DL Individual and environmental predictors of adjustment during the first year of college J College Stud Dev 1995 36 347 360

[B7] Stewart-Brown S Evans J Patterson J Petersen S Doll H Balding J Regis D The health of students in institutes of higher education: an important and neglected public health problem? J Public Health Med 2000 22 4 492 9 10.1093/pubmed/22.4.492 11192277

[B8] Wells J Barlow J Stewart-Brown S A systematic review of universal approaches to mental health promotion in schools Health Educ 2003 103 4 197 220 10.1108/09654280310485546

[B9] Johnston LD Bachman JG O’Malley PM Schulenberg JE The monitoring the future project after thirty-two years: design and procedure. (Monitoring the Future Occasional Paper No. 64) 2006 Ann Arbor, MI: Institute for Social Research

[B10] Wechsler H Kuo M College students define binge drinking and estimate its prevalence: Results of a national survey J Am Coll Health 2000 49 2 57 64 10.1080/07448480009596285 11016129

[B11] Gliksman L Adlaf EM Demers A Newton-Taylor B Heavy drinking on Canadian campuses Can J Public Health 2003 94 1 17 21 12583664 10.1007/BF03405045 PMC6980145

[B12] Schuit A Van-Loon J Tijhuis AJM Ocké M Clustering of lifestyle risk factors in a general adult population Prev Med 2003 35 219 24 12202063 10.1006/pmed.2002.1064

[B13] Rigotti NA Lee JE Wechsler H US college students’ use of tobacco products: results of a national survey JAMA 2000 284 669 705 10.1001/jama.284.6.699 10927777

[B14] American College Health Association (ACHA) Linthicum American College Health Association – National College Health Assessment II: Reference Group Executive Summary Fall 2009 2009 MD: American College Health Association

[B15] Cairney J Lawrence KA Smoking on campus: an examination of smoking behaviours among postsecondary students in Canada Can J Public Health 2002 93 4 313 6 12154537 10.1007/BF03405024 PMC6979770

[B16] Bray SR Born HA Transition to university and vigorous physical activity: implications for health and well-being J Am Coll Health 2004 52 4 181 8 10.3200/JACH.52.4.181-188 15018429

[B17] Bray SR Kwan MYW Physical activity is associated with better health and psychological well-being during transition to university J Am Coll Health 2006 55 2 78 82 10.3200/JACH.55.2.77-82 17017303

[B18] Kwan MYW Bray SR Martin Ginis KA Predicting physical activity during transition to first-year University: an application of the Theory of Planned Behaviour J Am Coll Health 2009 58 45 52 10.3200/JACH.58.1.45-55 19592353

[B19] Kwan MYW Arbour KP Lowe D Taman S Faulkner G Seeing may not believe: student reception, sources, and believability of health-related information J Am Coll Health 2010 58 555 62 10.1080/07448481003705925 20452932

[B20] Arbour-Nicitopoulos KP Kwan MYW Taman S Lowe D Faulkner GEJ Normative beliefs in health behavioural practices in a college population J Am Coll Health 2010 59 191 6 10.1080/07448481.2010.502194 21186449

[B21] American College Health Association The American College Health Association National College Health Assessment (ACHA-NCHA), Spring 2005 Reference Group Data (Abridged) J Am Coll Health 2006 55 5 16 16889310 10.3200/JACH.55.1.5-16

[B22] Laska MN Pasch KE Lust K Story M Ehlinger E Latent class analysis of lifestyle characteristics and health risk behaviours among college youth Prev Sci 2009 10 4 376 86 10.1007/s11121-009-0140-2 19499339 PMC2927491

[B23] Health Canada Eating Well with Canada's Food Guide 2007 Ottawa: Queen's Printer Available at: http://www.hc-sc.gc.ca/fn-an/food-guide-aliment/gen_prin-eng.php (Accessed on June 20, 2012)

[B24] Das P Horton R Rethinking our approach to physical activity The Lancet 2012 380 9838 189 90 10.1016/S0140-6736(12)61024-1 22818931

[B25] Johnston LD Bachman JG O’Malley PM Schulenberg JE Monitoring the Future Occasional Paper The monitoring the future project after thirty-two years: design and procedure 2006 64 Ann Arbor, MI: Institute for Social Research

[B26] Brener N Billy J Grady W Assessment of factors affecting the validity of self-reported health risk behaviour among adolescents: evidence from the scientific literature J Adolescent Health 2003 33 6 436 57 10.1016/S1054-139X(03)00052-1 14642706

[B27] Sloboda Z Sloboda Z Defining and measuring drug abusing behaviours Epidemiology of Drug Abuse 2005 New York: Springer

